# Complete genome sequence of the *Staphylococcus aureus* NCCP 11854 isolated from a food-poisoning patient in the Republic of Korea

**DOI:** 10.1128/mra.01101-24

**Published:** 2025-02-06

**Authors:** Joon Ki Kim, Chi-Hwan Choi, Mi-ran Yun, YeWon Ahn, Young Sill Choi, Su Yeon Kim

**Affiliations:** 1 Division of Pathogen Resource Management, Center for Vaccine Research, National Institute of Health, Korea Disease Control and Prevention Agency (KDCA), Cheongju, Republic of Korea; 2 Division of High-Risk Pathogens, Department of Laboratory Diagnosis and analysis, Korea Disease Control and Prevention Agency (KDCA), Cheongju, Republic of Korea; 3 Division of Infectious Disease Vaccine Research, Center for Vaccine Research, National Institute of Health, Korea Disease Control and Prevention Agency (KDCA), Cheongju, Republic of Korea; University of Maryland School of Medicine, Baltimore, Maryland, USA

**Keywords:** *Staphylococcus aureus*, NCCP 11854, CP168642.1, CP168643.1

## Abstract

*Staphylococcus aureus* is the etiological agent of skin diseases and food poisoning. In this study, we report the complete genome sequence of *S. aureus* NCCP 11854, which was originally isolated from the pus of a food-poisoning patient in the Republic of Korea in 2009.

## ANNOUNCEMENT

*Staphylococcus aureus* is a gram-positive, facultatively anaerobic, spherically shaped bacterium that is catalase-negative and coagulase-positive (1). *S. aureus* is a resident flora, but it can cause skin diseases, food poisoning, and respiratory infections as an opportunistic bacterium (2).

*Staphylococcus aureus* NCCP 11854 was selected as an alternative candidate for reference strain ATCC 6538 used in the Korean Pharmacopoeia (3), and genome sequencing was performed for genetic homogeneity analysis with the reference strain.

The strain was obtained from the National Culture Collection for Pathogens (NCCP) and cultured on 5% sheep blood agar (Synergy Innovation, Korea) for 18 h at 37°C. Genomic DNA was extracted by the phenol extraction and ethanol precipitation methods and was used for all experimental procedures. The strain was identified as *S. aureus* (99.7% identity) based on taxonomic classification of the 16S rRNA gene, obtained via Sanger sequencing with primers 27F (5′-AGAGTTTGATCMTGGCTCAG-3′) and 1492R (5′-TACGGYTACCTTGTTACGACTT-3′). Classification was performed using SINA (v1.2.12) (4) with the SILVA (v138.2) online database (5).

Genome assembly employed a hybrid approach combining long reads from PacBio RS II (Pacific Biosciences, Menlo Park, CA, USA) and short reads from Illumina MiSeq (Illumina, CA, USA).

For short-read sequencing, a paired-end sequencing library was prepared using Illumina DNA Prep kit (Illumina) according to the manufacturer’s instructions and sequenced on the MiSeq in 2 × 250 bp format using a MiSeq reagent kit v2 (Illumina). For long-read sequencing, additionally, gDNA was sheared using g-TUBES (Covaris, USA) followed by end-repair and SMRTbell adapters ligation. The library was prepared according to the manufacturer’s instructions using the SMRTbell Template Prep Kit 1.0 (Pacific Biosciences) and sequenced on the PacBio RS II with P6-C4 chemistry. This yielded a total of 96,094 long reads (1,005,348,493 bp; *N_50_* 10,462 bp), and 8,347,318 short reads (1,943,910,980 bp). Long reads (length <1,000 bp) were trimmed using Filtlong v0.2.1 (6) and short reads (removal of adapters, length <50 bp, and quality scores < 30) were processed using Fastp v0.23.4 (https://github.com/OpenGene/fastp). A total of 71,308 long reads (955,083,151 bp; *N_50_* 16,722 bp) and 6,362,680 short reads were used for assembly with Unicycler 0.5.0 (7), resulting in a circularized chromosome—rotated to start at the *dnaA* gene—and a plasmid that remained unrotated. Both assemblies were assessed using CheckM (v.1.2.2) (8) and annotated with the Prokaryotic Genome Annotation Pipeline (version 6.8) (9). Assembly statistics and data availability are summarized in Table 1. The strain, which contains no personal information, is available in the NCCP; therefore, institutional review board approval was not necessary for this research.

**TABLE 1:** Information and data availability of *S. aureus* NCCP 11854

	*Staphylococcus aureus* NCCP 11854
Genome accession IDs	CP168642.1 (chromosome), CP168643.1 (plasmid), PQ620151 (16S rRNA)
Sequence read archives	SRR30398399 (PacBio RS II), SRR24293587 (MiSeq)
Genome size (bp)	2,750,982 (chromosome), 17,697 (plasmid)
GC content (%)	32.9 (chromosome), 28.3 (plasmid)
Genome coverage	796× (chromosome), 2,589× (plasmid)
Completeness (%)	99.51
Contamination (%)	0.22
Number of predicted genes	2,710

## Data Availability

The accession numbers of sequences and the raw reads are included in Table 1.
